# Supplementary material for: The Rheology and Textural Properties of Bakery Products Upcycling Brewers’ Spent Grain
Source: Foods. 2023 Sep 22;12(19):3524. doi: 10.3390/foods12193524 (PMC10572393; doi:10.3390/foods12193524)
Supplement: Supplementary file 1 [file foods-12-03524-s001.zip › foods-2597823-supplementary.pdf]

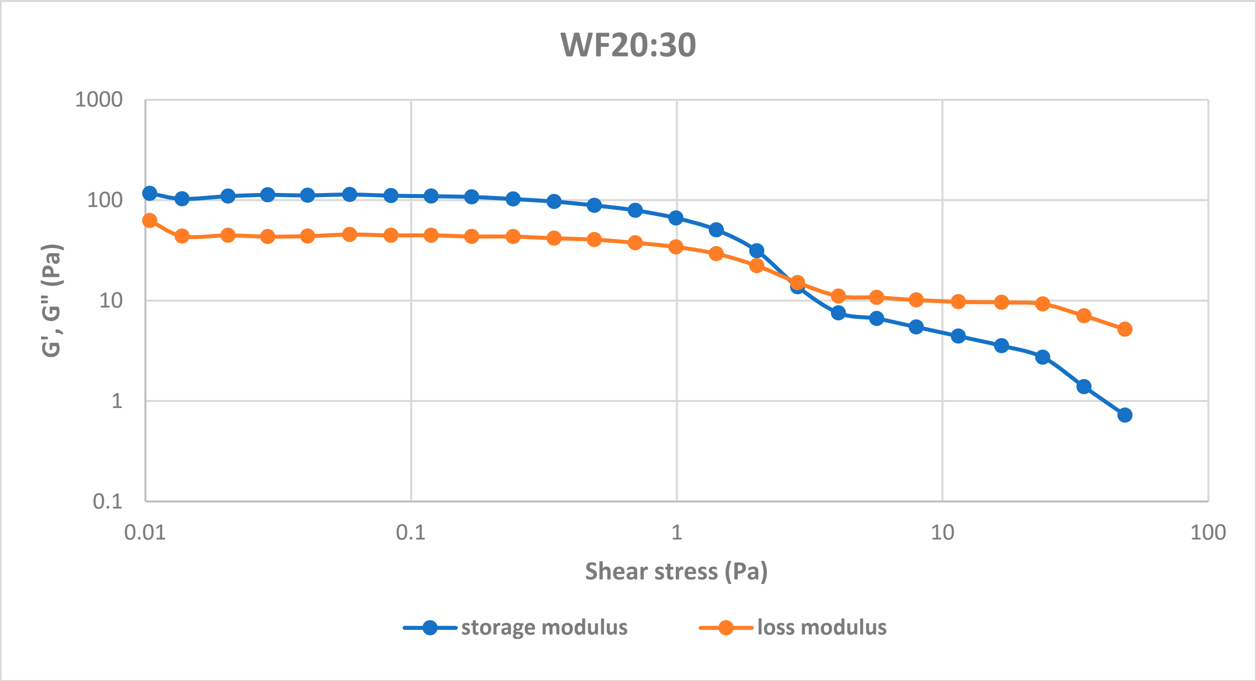

Figure S1a: Representative plots used for the amplitude assay of WF20:30 (= 20g wheat flour + 30g water)

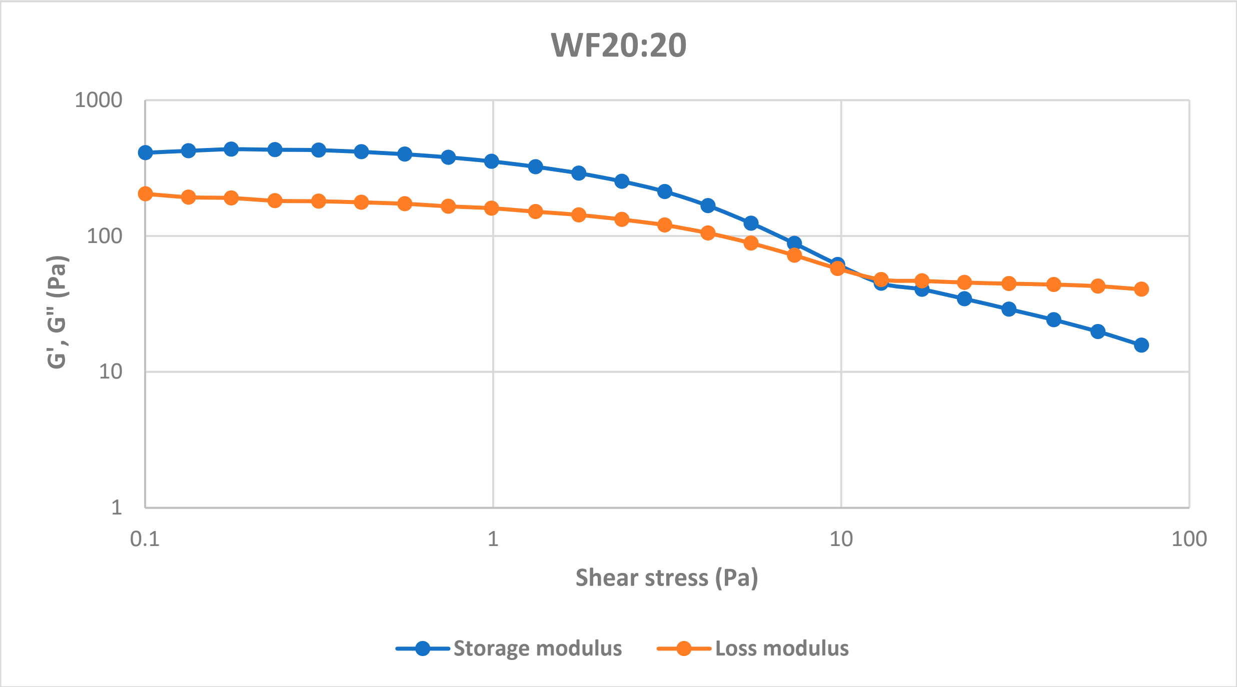

Figure S1b: Representative plot used for the amplitude assay of WF20:20 (= 20g wheat flour + 20g water)

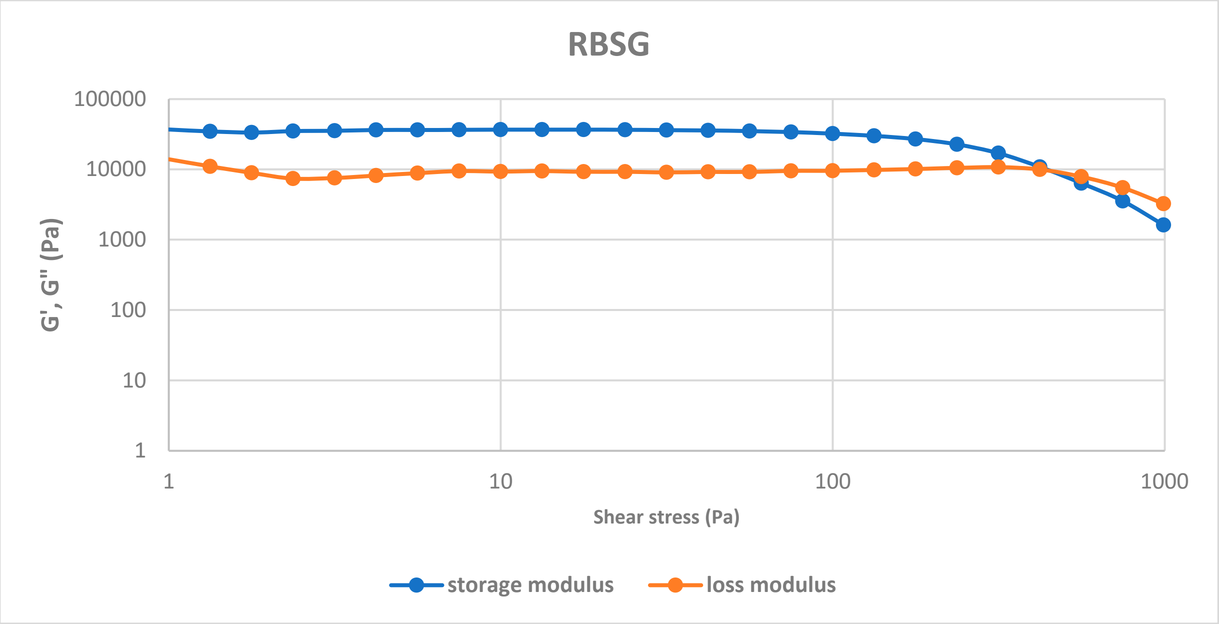

Figure S1c: Representative plot used for the amplitude assay of RBSG (=- 20g rye based BSG flour + 30g water)

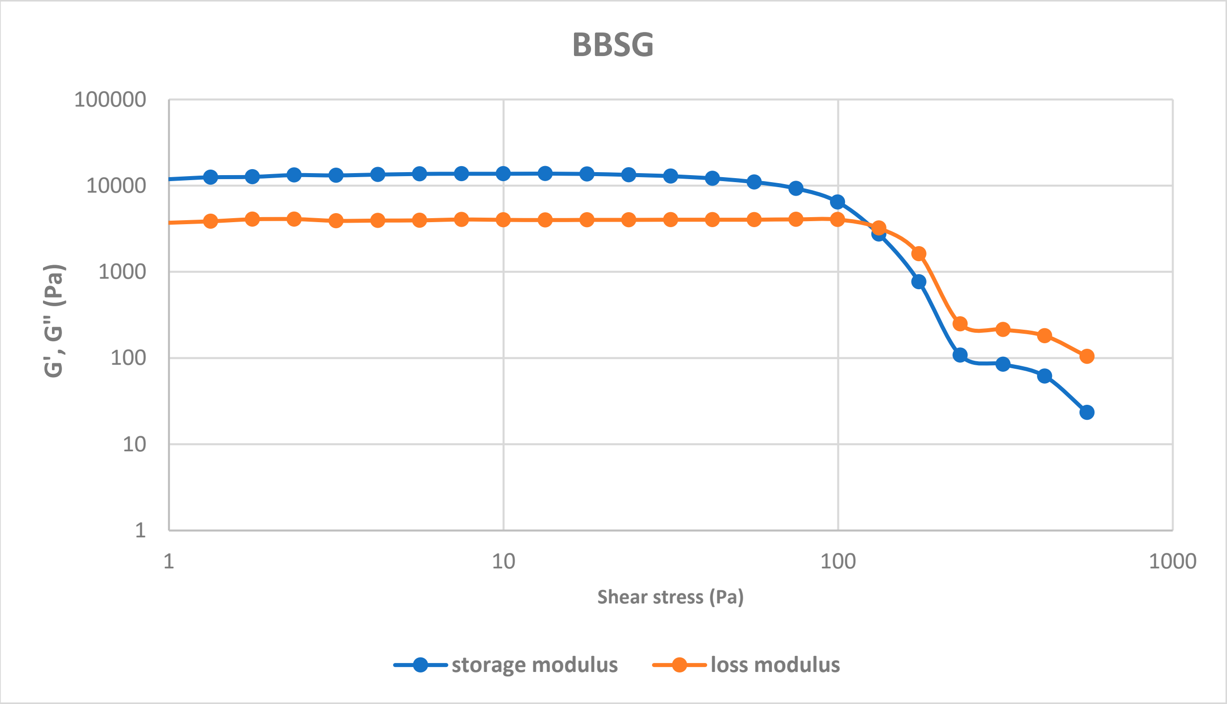

Figure S1d: Representative plot used for the amplitude assay of BBSG (= 20g barely based BSG flour + 30g water)

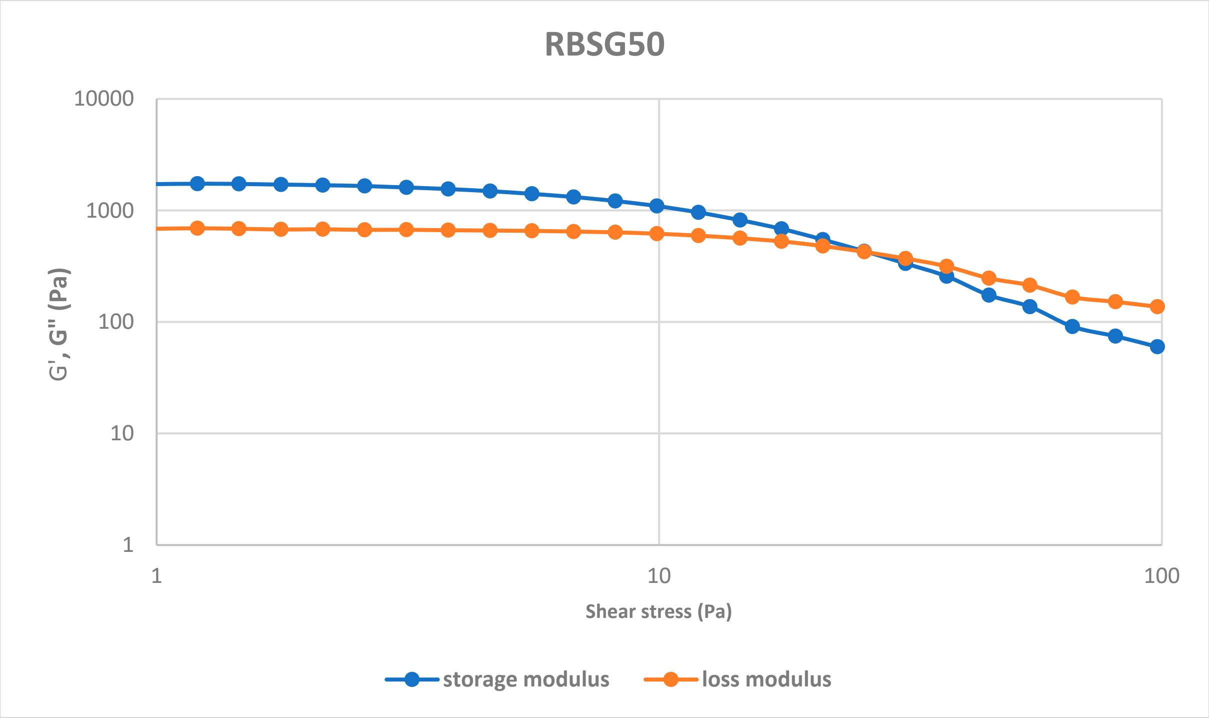

Figure S1e: Representative plot used for the amplitude assay of RBSG50 (= 10g wheat flour + 10g rye based BSG flour + 30 g water

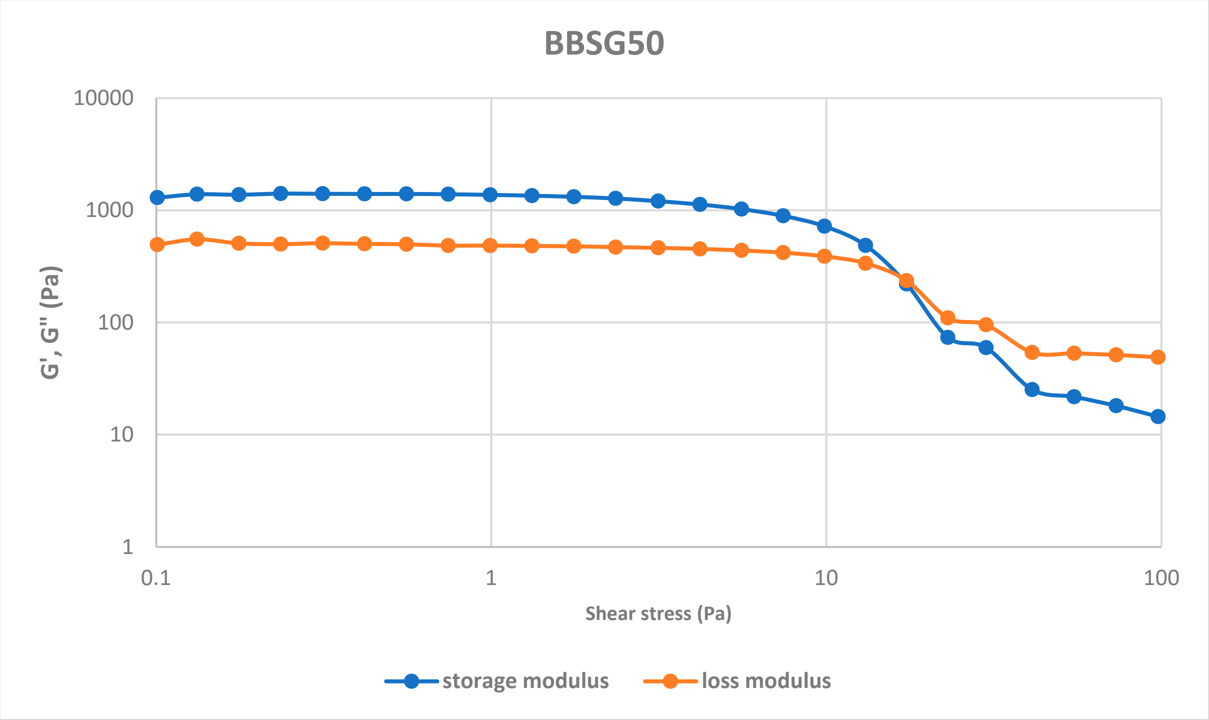

Figure S1f: Representative plot used for the amplitude assay of BBSG50 (= 10g wheat flour + 10g barley BSG flour + 30 g water).

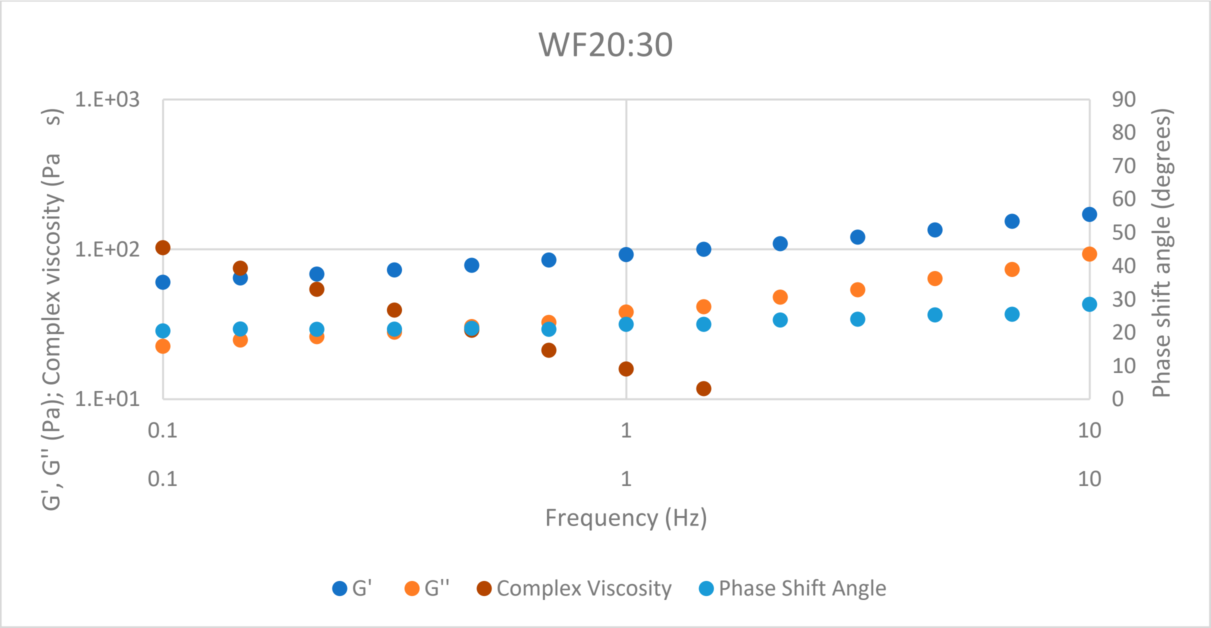

Figure S2a: Representative mechanical spectra of frequency sweep of WF20:30 (= 20g wheat flour + 30g water)

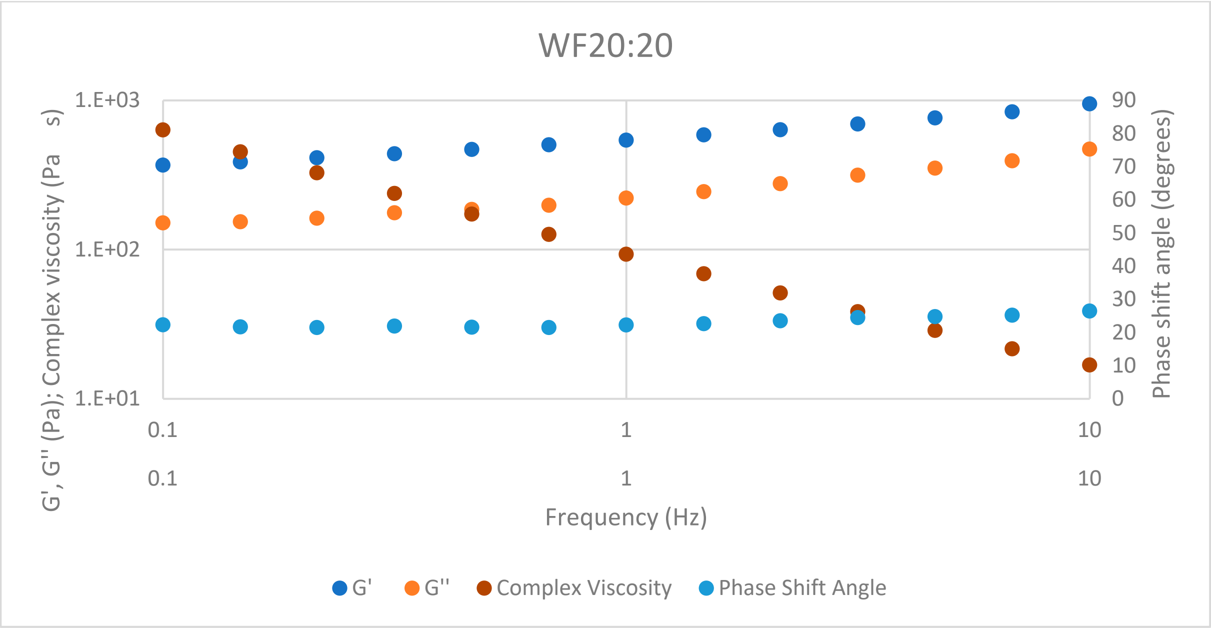

Figure S2b Representative mechanical spectra of frequency sweep of WF20:20 (= 20g wheat flour + 20g water)

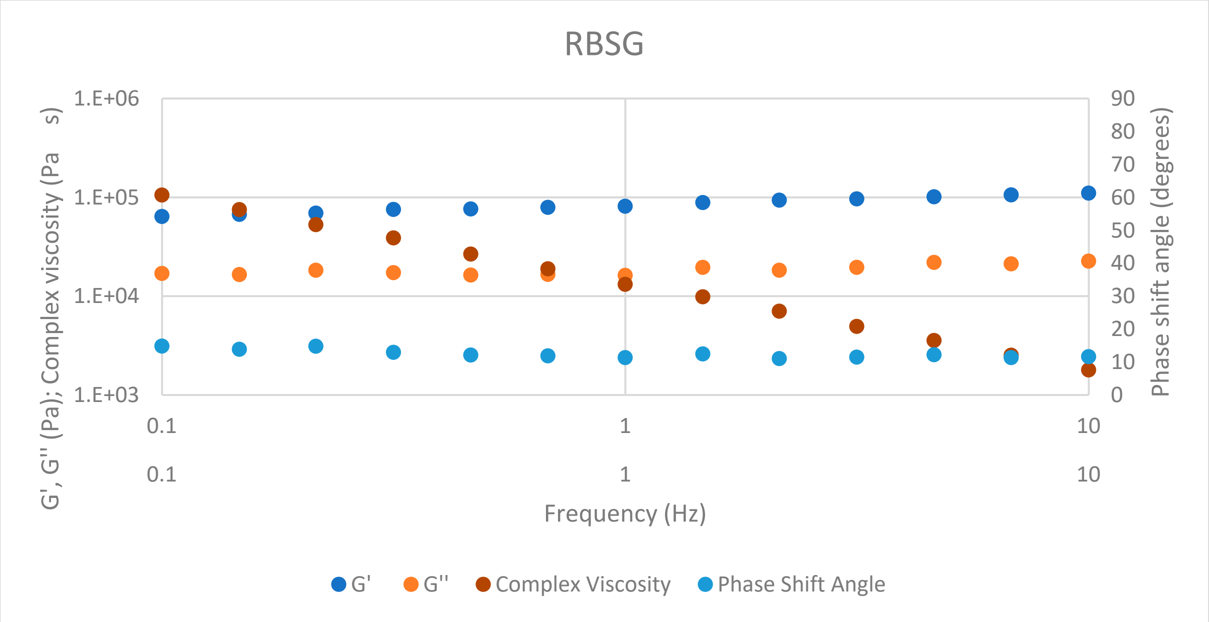

Figure S2c: Representative mechanical spectra of frequency sweep of RBSG (=- 20g rye based BSG flour + 30g water)

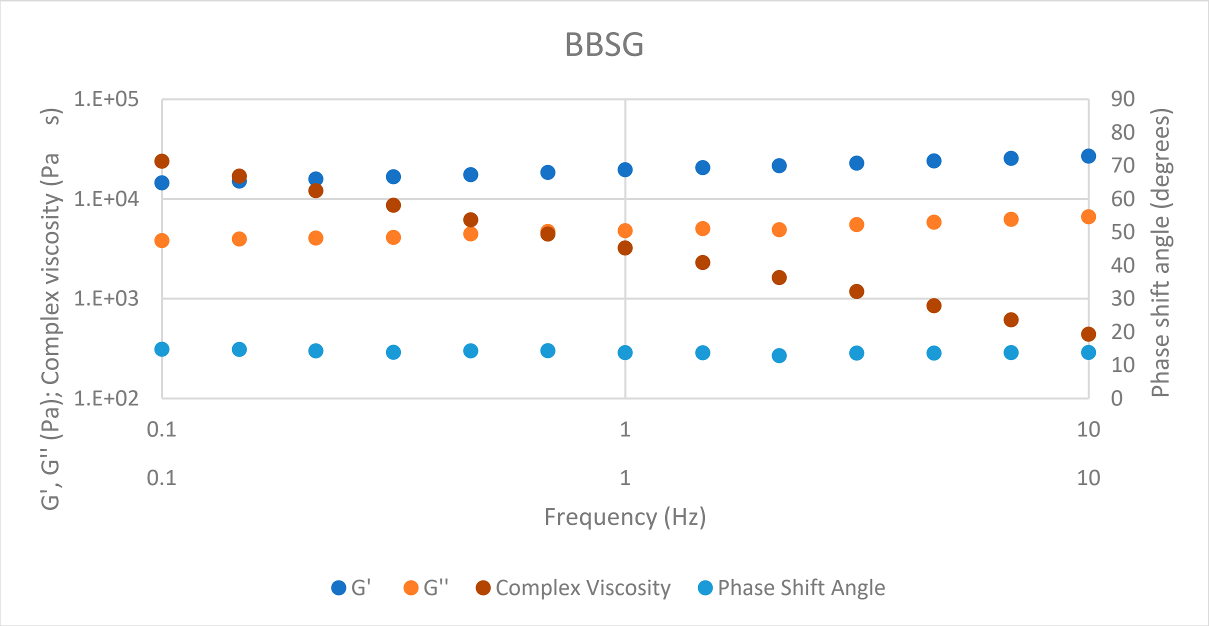

Figure S2d: Representative mechanical spectra of frequency sweep of (= 20g barely based BSG flour + 30g water)

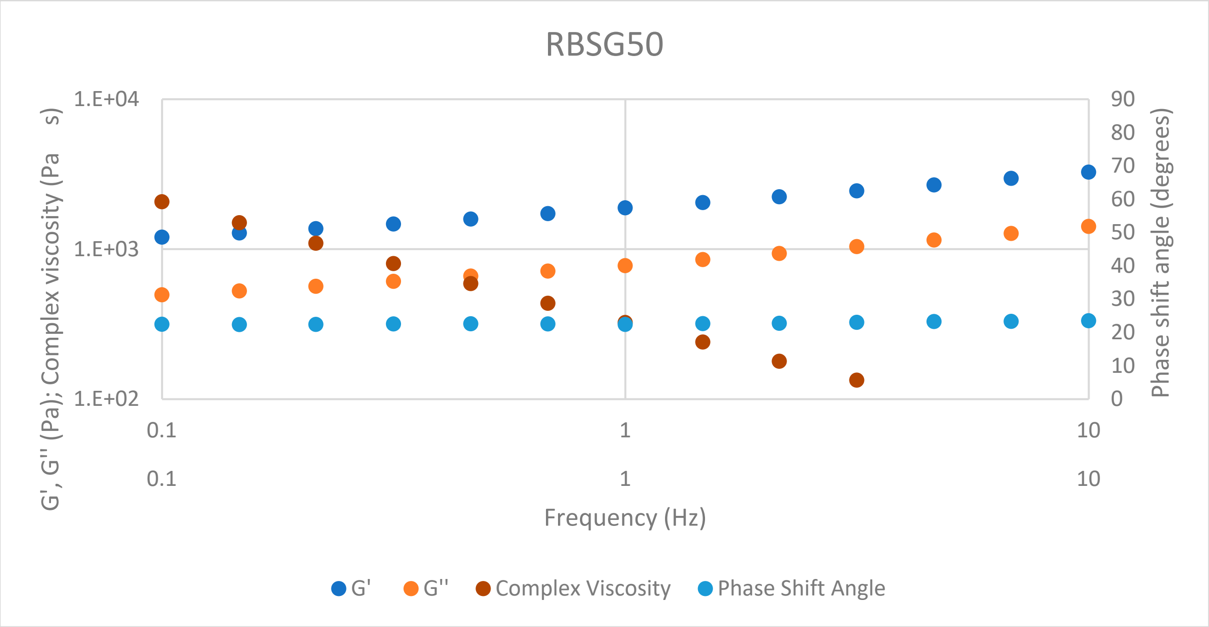

Figure S2e: Representative mechanical spectra of frequency sweep of RBSG50 (= 10g wheat flour + 10g rye based BSG flour + 30 g water

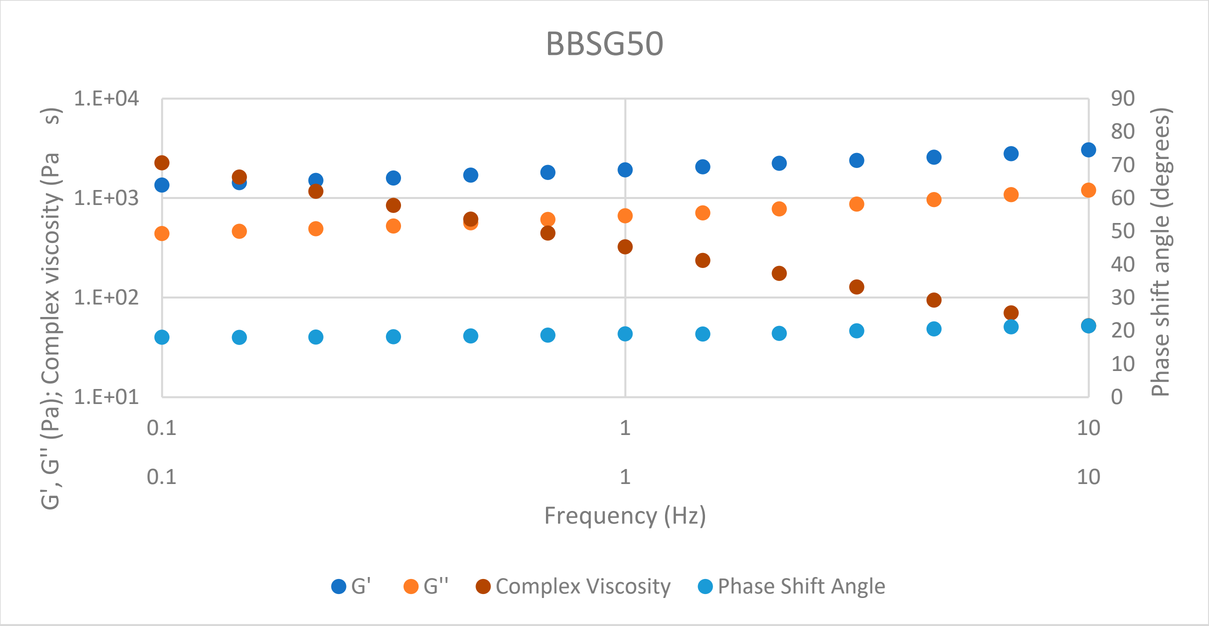

Figure S2f: Representative mechanical spectra of frequency sweep of BBSG50 (= 10g wheat flour + 10g barley BSG flour + 30 g water).
